# Applicability of Additives for Ground Improvement Utilizing Fine Powder of Waste Glass

**DOI:** 10.3390/ma14185169

**Published:** 2021-09-09

**Authors:** Shinya Inazumi, Ryo Hashimoto, Takashi Shinsaka, Supakij Nontananandh, Susit Chaiprakaikeow

**Affiliations:** 1 Department of Civil Engineering, Shibaura Institute of Technology, Toyosu Campus, Tokyo 135-8548, Japan; 2 Doboku Chishitsu Co. Ltd., Sendai 981-3107, Japan; rhashimoto@geoce.co.jp; 3 Sanshin Corporation, Tokyo 111-0052, Japan; shinsaka@sanshin-corp.co.jp; 4 Department of Civil Engineering, Kasetsart University, Chatuchak, Bangkok 10900, Thailand; fengskn@ku.ac.th (S.N.); fengssck@ku.ac.th (S.C.)

**Keywords:** additive, fine powder of blast furnace slag, fine powder of waste glass, ground improvement, solidifying material

## Abstract

As a solidifying material for ground improvement using inorganic waste as a raw material, the authors have been developing an additive mixture of the fine powder of waste glass containing a large amount of silica generated during the production of glass cullet and an alkaline aid (heat-treating type of “Earth-Silica; ES” additive). Furthermore, a solidifying material that solidifies by mixing this additive with the fine powder of blast furnace slag, which is a by-product of steel production, is also being developed. In this study, the authors reviewed the mixing process of the solidified materials, especially the one made with the heat-treating type of ES additive, omitting the heat treatment of the fine powder of waste glass and the alkaline aid and applying only the mixing treatment. As a result, a mixing type of ES additive was manufactured to simplify the manufacturing process, and the difference in the performance of the solidifying material, depending on the presence or absence of the heat-treating process during the additive manufacturing, was verified in terms of the effect on the solidifying action. Specifically, the solidifying materials to which the heat-treating type of ES additive and the mixing type of ES additive were added, respectively, were applied to the high-pressure injection stirring method, one of the ground-improvement methods. Various tests clarified the changes in viscosity of these solidifying materials over time and the acceleration of their solidifying rates when adding ordinary Portland cement separately.

## 1. Introduction

The sustainable development of industries is desired in modern society, and giving consideration to the environment is becoming more and more important with the progress of technologies. In the case of glass, one kind of industrial product, it is collected after its use and reused as a raw material for glass, but the fine powder generated during the production of glass cullet is landfilled as general waste [1,2,3]. As a result, in Japan alone, 2.82 million tons of this fine powder, or 25% of the amount of non-ferrous metal mineral waste including glass waste, was not recycled, but discarded in final disposal sites, such as landfills, in 2017 [4,5,6].

In a series of studies [7,8,9,10], the authors developed a solidifying material for ground improvement that contributes to a reduction in the amount of waste treatment by utilizing inorganic waste as a starting material. This solidifying material is composed of the fine powder of blast furnace slag and an additive developed independently. About 40% of the additive is the fine powder of waste glass containing a large amount of silica as the starting material, which is mixed with a powdery alkaline aid, heat-treated, and pulverized by pulverization (hereinafter, the “heat-treated type of ES additive” in which ES means Earth-Silica). The fine powder of blast furnace slag is pulverized blast furnace granulated slag produced as a by-product in steel production. The characteristics of this solidifying material include the following items [7,8,9,10]:

(a) Development of very high strength can be expected even when the target soil is cohesive soil.
(b) Even after 28 days of curing, a large increase in strength can be expected.
(c) By the fine tuning of the composition, the time at which solidification starts can be controlled.

However, a problem arises with solidifying material that has the above characteristics, namely, the manufacturing process of the heat-treating type of ES additive is complicated and the powdery alkaline aid becomes scattered during its use. Therefore, the heat treatment and pulverization treatment were omitted in the manufacturing process to simplify the manufacturing. In addition, while reviewing this powdery alkaline aid for the purpose of suppressing the scattering, the authors developed an additive that can be manufactured only by mixing. Hereinafter, this additive will be referred to as the “mixing type of ES additive”.

In this study, the mixing type of ES additive is used as a solidifying material in combination with the fine powder of blast furnace slag and is compared with the cement-based solidifying material, similar to the conventional heat-treating type of ES additive that requires both heat treatment and fine powder treatment [7,8,9,10]. Various laboratory tests were conducted on the specimens in which each mixing type of ES additive with the fine powder of blast furnace slag and cement-based solidifying material were mixed, respectively, with the prescribed composition for sandy soil and cohesive soil, and then they were compared.

## 2. Challenges of Stirring System Consolidation Methods

In examining the applicability of the solidifying material, stirring system consolidation methods, comprising one of the ground-improvement methods, will be described.

Stirring system consolidation methods are ground-improvement methods in which a solidifying material and the ground are agitated and mixed to bring about solidification. Typical methods include the mechanical stirring method and the high-pressure injection stirring method.

In the mechanical stirring method, a stirring shaft with a stirring device (stirring blade) is penetrated into the ground, and the stirring blade is rotated while the solidifying material is being discharged in order to stir and mix the solidifying material and the original ground to improve the ground.

The high-pressure injection stirring method injects a slurry-like solidifying material, in a high-pressure state, into the ground with high-pressure compressed air. It stirs and mixes the solidifying material and the original ground while cutting the ground in order to strengthen it. During the ground improvement, part of the mixture of the cut raw ground and the solidifying material is discharged to the ground as mud, such that the ground improvement has little effect on any adjacent structures.

Regarding the mechanical stirring method and the high-pressure injection stirring method, the following items are reported [11,12,13,14,15,16,17].

### 2.1. Decrease in Liquidity

A decrease in fluidity is a phenomenon in which the viscosity increases sharply when the solidifying material and the raw ground are agitated and mixed. When the fluidity decreases, the mechanical stirring method cannot sufficiently stir and mix the solidifying material and the raw ground, and the variation in strength in the improved soil becomes large. In addition, there is a concern that problems, such as the inability to pull out the stirring device (stirring blade), may occur. With the high-pressure injection stirring method, not only does the variation in strength in the improved soil become large, but also the mud is blocked and the air and the mud cannot be recovered. In this case, not only does the quality of the improved soil deteriorate, but the influence on the surrounding ground and structures, due to the increase in pressure inside the ground by the blockage, increases [14].

Decreased fluidity often occurs in cohesive soil with a large amount of fine particles [11,13,15]. The reason for the decrease in fluidity is that cohesive soil with a large amount of fine particles contains a large amount of clay minerals with a large cation exchange capacity, and the Ca^2+^ in the solidifying material is adsorbed to form a network of aggregates.

Examples of methods for preventing the decrease in fluidity include the addition of a fluidizing agent and the use of a highly fluid type of solidifying material. However, if a large amount of fluidizing agent is used, solidification failure may occur.

### 2.2. Insufficient Strength in Cohesive Soil

As the soil particles in cohesive soil have a smaller diameter and more voids than those in sandy soil, a large amount of solidifying material is required to bond the soil particles to each other. However, due to the high viscosity of cohesive soil, it becomes difficult for the solidifying material to disperse to every corner of the voids. Therefore, the strength of the improved soil tends to be lower in cohesive soil than in sandy soil [11,13,15].

The idea of increasing the amount of solidifying material in order to counteract the lack of strength in cohesive soil is conceivable. If the amount of solidifying material is increased excessively, however, the viscosity of the mixture and the waste mud becomes high (the fluidity decreases) and, as described above, sufficient stirring and mixing cannot be performed. As a result, the quality may deteriorate and it may not be possible to perform stirring with the mechanical stirring method. In addition, the high-pressure injection stirring method may cause the obstruction of mud.

### 2.3. Delayed Performance in Overlapping Construction

When performing block-type improvement, wall-like improvement, or grid-like improvement, the basic arrangement is that adjacent improved soil columns overlap each other [16]. In the mechanical stirring method, when adjacent improved soil columns are constructed continuously, overlapping construction is possible. However, when there are intervals of days in the construction of adjacent improved soil columns, there will be a delay in the strength development of the columns when a solidifying material or retarding agent is used [12,17]. If the construction of adjacent improved soil columns is significantly delayed by unseasonable weather or construction troubles, the construction may be defective due to the progress of the hardening of the preceding improved soil columns. On the other hand, if an excessive amount of retarding agent is added, poor solidification may occur.

## 3. Materials and Methods

Based on the above-mentioned issues, the authors focused on the fluidity, strength characteristics, and delay effect of the solidifying materials themselves and the soils improved with the solidifying materials, and they conducted interior mixing tests to confirm the applicability of the mixing type of ES additive to the ground-improvement method. In addition, the authors also compared the performance with the heat-treating type of ES additive [7,8,9,10], which involves heat treatment and pulverizing in the manufacturing process that has been tested in the past.

### 3.1. Specimen Mixing

The interior mixing tests were performed according to the contents of Table 1. The solidifying material was a mixture of the heat-treating type of ES additive and the fine powder of blast furnace slag (hereinafter, A-type), which requires heat treatment and pulverization in the manufacturing process, and a mixture of the mixing type of ES additive and the fine powder of blast furnace slag (hereinafter, B-type). In addition, the cement-based solidifying material (hereinafter, C-type) used in the high-pressure injection stirring method was applied for comparison.

Based on the previous tests [7,8,9,10], the solidifying material of the A-type has a slow initial speed of solidification, and early solidification is achieved by partially replacing the fine powder of blast furnace slag with ordinary Portland cement (hereinafter, ordinary cement). This study also compared and verified whether the solidifying material of the B-type had the same properties as above.

Each composition of solidifying materials used in this study will be described. The heat-treating type of ES additive used for the A-type was a white powder with a SiO_2_ content of approximately 50% and a density of 2.56 g/cm^3^. On the other hand, the mixing type of ES additive used for the B-type was a grayish white powder with a SiO_2_ content of approximately 55% and a density of 2.58 g/cm^3^, which is higher than that of the A-type. There was a slight difference in their content ratios. For the fine powder of blast furnace slag, JIS A 6206: 2013: Ground Granulated Blast Furnace Slag for Concrete (fine powder of blast furnace slag 4000) without dihydrate gypsum was used, with a density of 2.91 g/cm^3^, a specific surface area of 4400 cm^2^/g, and a vitrification rate of 99%. This fine powder of blast furnace slag was common to both the A-type and the B-type. The blending ratio of the fine powder of blast furnace slag (SL) to the heat-treating type of ES additive or mixing type of ES additive (H) was based on the weight ratio SL/H = 10, which is the standard blend for the A-type. However, for the purpose of early solidification, a mixture, in which the fine powder of blast furnace slag was replaced with ordinary cement (N) by 10% or 20% by weight, was also prepared (hereinafter, A-10-type, A-20-type, B-10-type, and B-20-type, respectively). The ordinary cement used here is of JIS (Japanese Industrial Standard), and the replacement ratio was converted to a weight ratio. In addition, in the interior mixing tests, all the solidifying materials were used in the state of a slurry solution with water added, and the weight ratio of the kneading water (W) to the solidifying material (B) was unified to W/B = 130%. The composition of the solidifying material here was the amount of cement-based solidifying material (C) (B = C) in the case of the C-type, and included ordinary cement being substituted for the A-type and the B-type solidifying materials. The total amount of each (B = H + SL or B = H + SL + N) was used.

**Figure 1:**
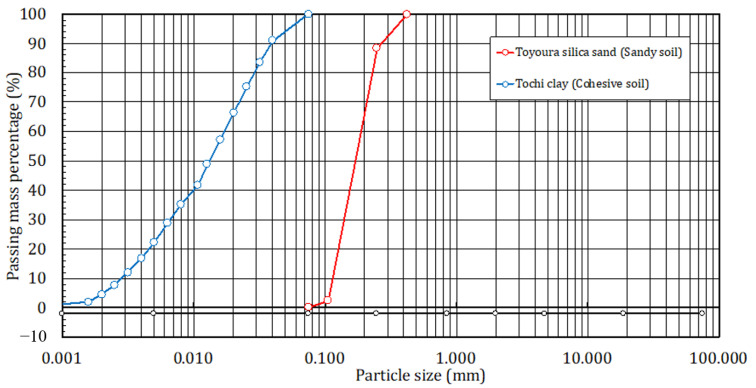
Grain size addition curve of target soils.

**Figure 2:**
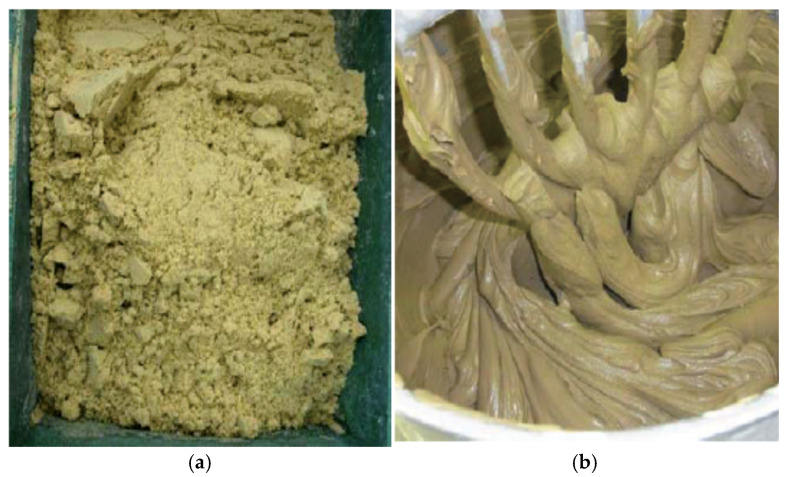
State of the target soil after adjusting the water content. (**a**) Toyoura silica sand (sandy soil). (**b**) Tochi clay (cohesive soil).

Figure 1 shows the particle size distribution curves for the sandy soil and the cohesive soil of the target soil used as the ground material, and Figure 2 shows the state of the target soil after adjusting the water content. The sandy soil used for the ground material was Toyoura silica sand (ρ_s_ = 2.63 g/cm^3^) collected from the Toyoura area of Yamaguchi Prefecture, Japan. It was used after adjusting the water content to w = 10%. The cohesive soil was Tochi clay (ρ_s_ = 2.65 to 2.70 g/cm^3^) collected from the Aso area of Tochigi Prefecture, Japan. The water content was adjusted to w = 40%, which is slightly higher than the liquid limit (w_L_ = 34%). The Tochi clay was used after being left standing for a period of 12 h or more to allow it to become completely combined with the water. The mixing ratio of the target soil (S) and the slurry solution (M = W + B) was unified to S/M = 2.0 in terms of the volume ratio in all the proportions.

The approximate value of the quantity of each material per 1 m^3^ of target soil, in the case of an actual on-site construction, is as follows:

- Slurry solution (M): volume 0.5 m^3^ and mass 700 kg;
- Water (W): 390 kg;
- Solidifying material (C or H+SL or H+SL+N): 310 kg;
- Fine powder of blast furnace slag (including replaced cement) (SL or SL+N): 280 kg;
- ES additive (H): 30 kg.

The specimen preparation process was carried out in the same manner as in [7,8,9,10] by the following procedure.

(1) When replacing a portion of the fine powder of blast furnace slag with cement, mix the fine powder of the blast furnace slag and the cement powder and stir with a mixer for 30 s.
(2) Weigh and add water to the fine powder of the blast furnace slag (or fine powder of blast furnace slag + cement), and stir with a mixer for 1 min.
(3) Weigh and add the ES additive and stir with a mixer for 3 min. (Slurry solution is used for the P-funnel test.)
(4) Weigh and mix the slurry solution and the target soil, and stir with a mixer for 3 min. (Mixed specimen is used for the cylinder method flow test.)
(5) Fill the molded container with the mixed specimen. (After the material has reached the specified age, it is used for the unconfined compression test.)
(6) In the case of the cement-based solidifying material (C-type), follow Steps (1) and (2) for mixing and stirring the water and the cement.

### 3.2. Test Methods

In the interior mixing tests, when examining the applicability of the solidifying material, the verification items are the tendency of the fluidity of the slurry solution to change over time, the initial fluidity of the mixed specimen of the target soil and the slurry solution, and the strength of each material after curing. In each interior mixing test, three specimens are prepared under each test condition and subjected to the test, and the average of the three results is used as the representative value for each test and each condition.

#### 3.2.1. Fluidity Test (P-Funnel Test)

In the high-pressure injection stirring method, when the slurry solution is injected using a nozzle, the nozzle may become blocked if the slurry solution is highly viscous; thus, it is necessary to verify the fluidity of the slurry solution. To verify the fluidity, it was evaluated according to the Japan Society of Civil Engineers (JSCE) standard “Fluidity Test Methods for Injected Mortar of Prepacked Concrete (Method by P-Funnel)” (JSCE-F521). This is a test in which a slurry solution, prepared by mixing kneading water and a solidifying material, is poured into a P-funnel immediately after kneading and, after 3 h have passed, the flow time of the slurry solution filled to a specified amount is measured. At this time, the higher the viscosity of the slurry solution, the longer it takes to flow down from the P-funnel. The flow-down time measurement after the 3 h has passed takes into consideration the delay in work that occurs under various conditions during construction.

#### 3.2.2. Flow Test

The purpose of the flow test is to confirm the ease with which the improved soil can be stirred during the mechanical stirring method in the cohesive soil, and to confirm the fluidity of the wastewater generated by the high-pressure injection stirring method. An evaluation of the fluidity was carried out according to the consistency test (hereinafter, cylinder method) of the Nippon Expressway Company Limited (NEXCO) standard “Test method for air mortar and air milk (cylinder method)” (JHSA313), using a specimen obtained by stirring and mixing a solidifying material in the form of a slurry solution and cohesive soil.

This test uses a hard plastic cylinder, with a size of ϕ80 mm and L80 mm, fills the mixed specimen, and confirms the fluidity by the diameter (flow value) at which the material spreads when the cylinder is pulled up. At this time, the larger the flow value, the higher the fluidity of the material.

#### 3.2.3. Unconfined Compression Test

The required number of specimens was prepared by stirring and mixing the solidified material in the form of a slurry solution. The target soil was placed in a cylindrical container mold with a size of ϕ50 mm × L100 mm. The material ages (curing times) were 1, 7, 28, 56, and 91 days, and the strength at each material age was measured by an unconfined compression test. If solidifying was not observed within 1 or 7 days of the initial age of the material, the strength development was evaluated using the age of the material from the stage when it was cured to the state when it could stand on its own at the time of demolding as the initial strength.

## 4. Results and Discussion

### 4.1. Fluidity Test (P-Funnel Test)

Table 2 shows the results of fluidity tests using slurry solutions of various solidifying materials. As shown by the values in this table, there is almost no difference in viscosity among the slurry solutions with the A-type, B-type, and C-type, respectively. Even after 3 h, the flow time is approximately 9 s. The slurry solution of the B-type, developed this time, is seen to have the same level of fluidity as the slurry solution of the A-type, already developed, as well as that of the conventional cement-based slurry solution. Therefore, the slurry solution of the B-type can be applied to both the mechanical stirring method and the high-pressure injection stirring method.

### 4.2. Flow Test

**Figure 3:**
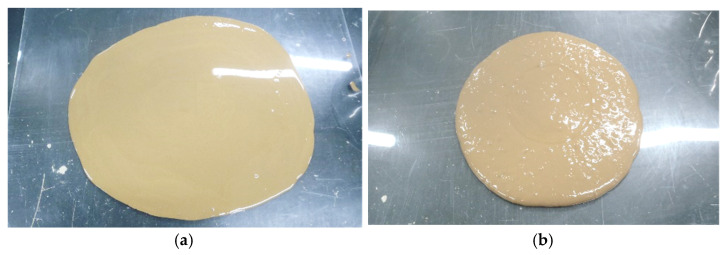

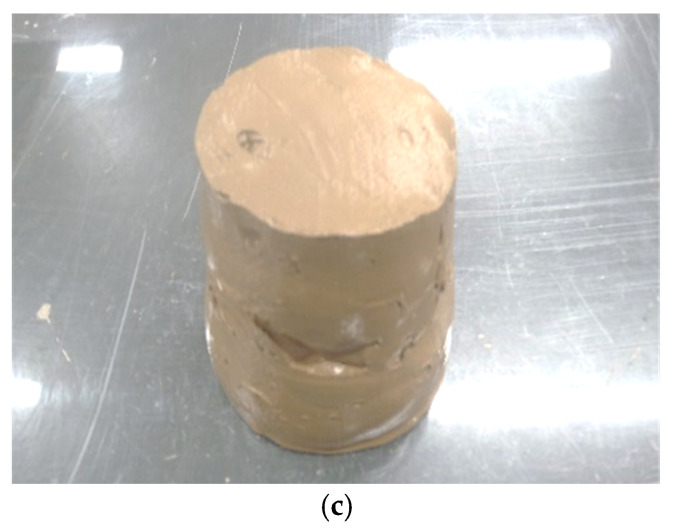
State of the flow test for specimens prepared from mixed samples of cohesive soil and slurry solution of each solidifying material. (**a**) B-type (equivalent to No. 11 in Table 3). (**b**) B-20-type (equivalent to No. 13 in Table 3). (**c**) C-type (equivalent to No. 14 in Table 3).

Table 3 shows the results of the flow tests for the specimen mixed with cohesive soil and the slurry solution of each solidifying material, and Figure 3 shows an example of the flow test of the specimen. From the results of the flow test, the flow value of the C-type is smaller than that of the solidifying materials of both the A-type and the B-type, and the specimen does not spread much even when the cylinder of ϕ80 mm is pulled up. On the other hand, high fluidity was confirmed for both the A-type and the B-type, with the results revealing higher flow values for the B-type. In addition, the A-type and the B-type also have a common tendency for the flow value to decrease as the replacement ratio of ordinary cement increases, and there is no significant difference in characteristics between the two. The fluidity of the newly developed B-type and the cohesive soil in a mixed state is equivalent to that of the already developed A-type.

Like the A-type, the B-type has much higher fluidity than the C-type after mixing with cohesive soil. In other words, in the process of ground improvement, it is possible to construct a high-quality improved soil body with little variation. With the mechanical stirring method, it is possible to operate a construction machine with a small stirring force, while with the high-pressure injection stirring method, it is possible to suppress the blockage due to mud drainage, and it is considered that the influence on the surrounding soil (ground) is reduced.

### 4.3. Unconfined Compression Test

#### 4.3.1. Sandy Soil

**Figure 4:**
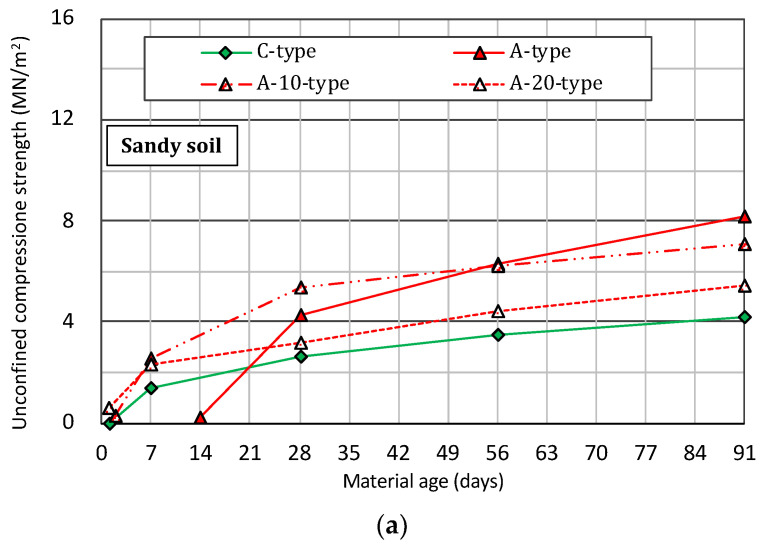

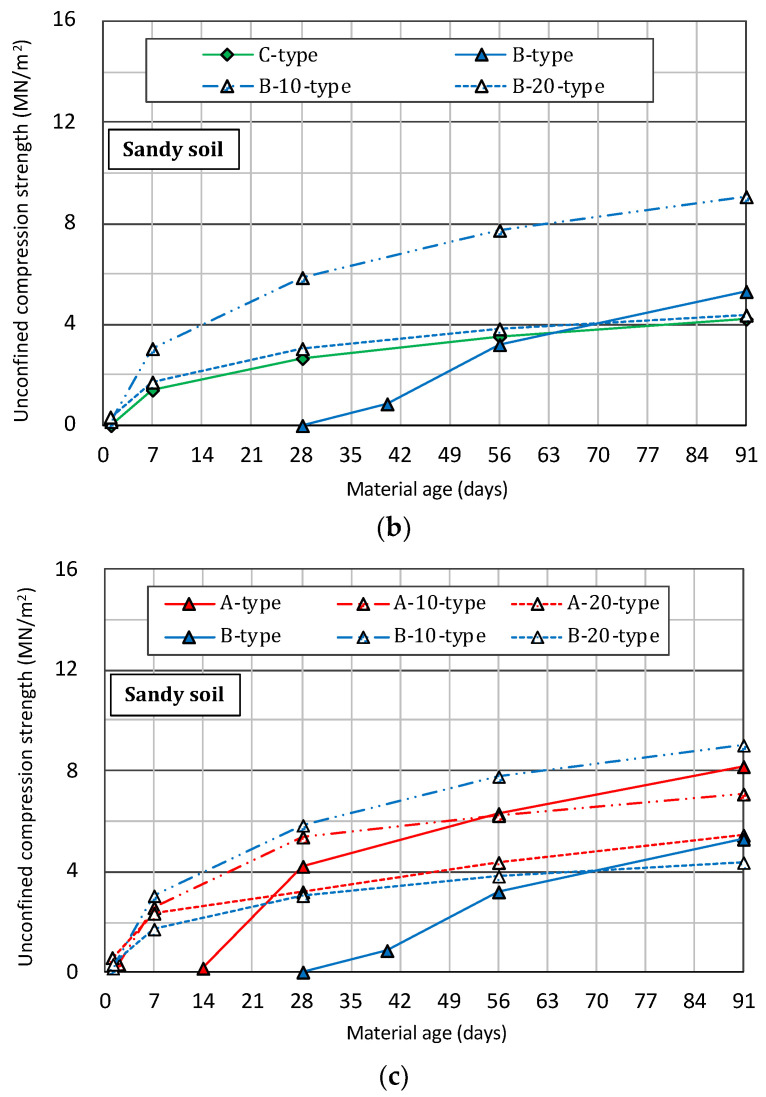
Changes over time in the unconfined compression strength of the specimen prepared by mixing sandy soil and a slurry solution of various solidifying materials with the passage of the material age. (**a**) A-type and C-type. (**b**) B-type and C-type. (**c**) A-type and B-type.

Figure 4 shows the changes over time in the unconfined compression strength of the specimen prepared by mixing sandy soil and a slurry solution of various solidifying materials with the passage of the material age. From Figure 4a, in the case of the A-type, the unconfined compression strength for the long-term material age of 28 days is 4 to 8 MN/m^2^ or more compared to 3 to 4 MN/m^2^ for the C-type. That is, higher strength development was confirmed in the case of the A-type. However, for the early material age of less than 28 days, the A-type has a small unconfined compression strength and a slow onset of strength. On the other hand, when the fine powder of blast furnace slag is replaced with ordinary cement by 10% or 20% by weight (A-10-type or A-20-type), in anticipation of strength development at an early material age, the unconfined compression strength of both the early and the long-term material ages is equal to or higher than that of the C-type.

In the strength comparison between the B-type and the C-type shown in Figure 4b, the strength development of the B-type mixture is relatively slow. On the other hand, like the A-type, the B-type develops strength relatively early when the fine powder of blast furnace slag is replaced with ordinary cement by 10% or 20% by weight (B-10-type or B-20-type). Furthermore, in the mixture with a 10% substitution of ordinary cement (B-10-type), the strength was 9 MN/m^2^ at the material age of 91 days, which is more than double the strength of the C-type.

In the strength comparison between the A-type and the B-type, shown in Figure 4c, a slight predominance can be seen in the strength development of the A-type. However, for the mixture in which the fine powder of blast furnace slag is replaced with ordinary cement (B-10-type or B-20-type), even if it is the B-type, it can be applied as a solidifying material having almost the same performance as the A-type.

#### 4.3.2. Cohesive Soil

**Figure 5:**
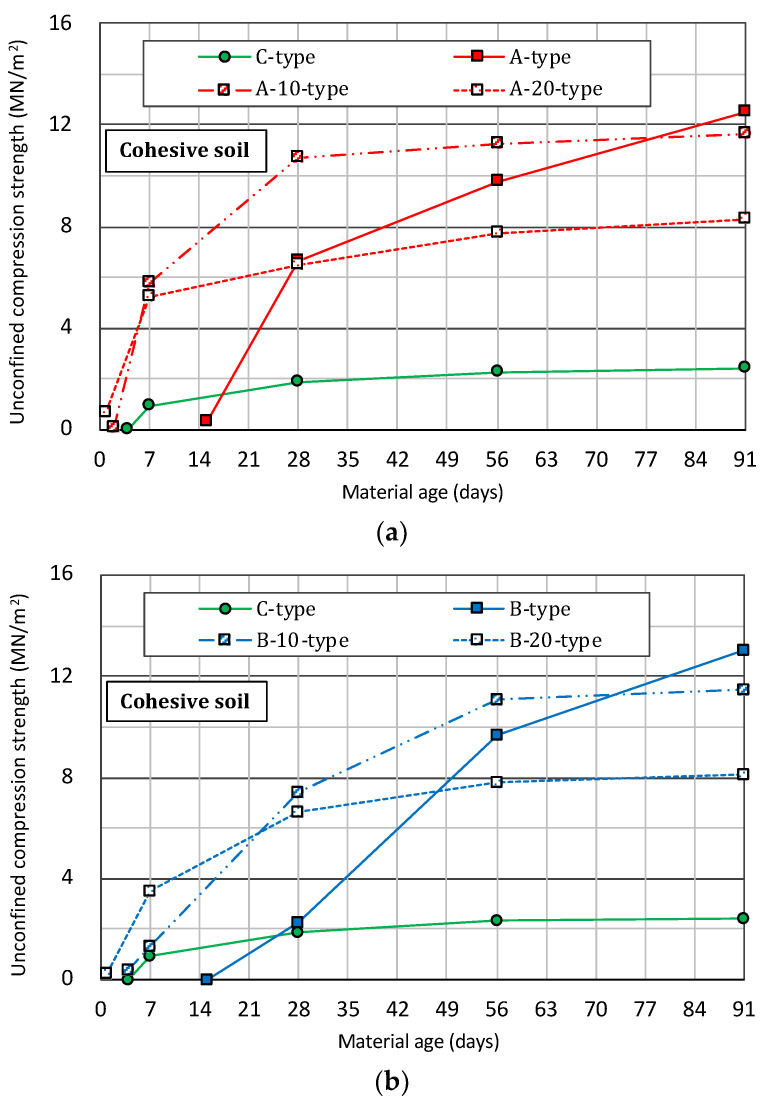

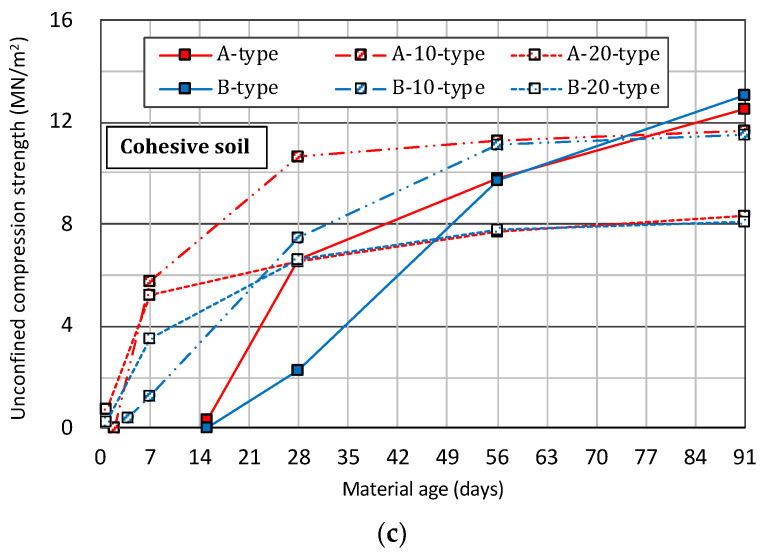
Changes over time in the unconfined compression strength of the specimen prepared by mixing cohesive soil and a slurry solution of various solidifying materials with the passage of the material age. (**a**) A-type and C-type. (**b**) B-type and C-type. (**c**) A-type and B-type.

Figure 5 shows the transition of unconfined compression strength with the passage of the material age in the specimen prepared by mixing cohesive soil and slurry solutions of various solidifying materials. In the strength comparison between the A-type and the C-type shown in Figure 5a, it can be confirmed that the strength development is slower in the A-type than in the C-type, as in the case of sandy soil. However, the strength at the material age of 28 days is 6 MN/m^2^ or more compared to 2 MN/m^2^ of the C-type, which is more than 3 times the strength. It is confirmed that the A-type mixture, in which 10% or 20% by weight of the fine powder of blast furnace slag is replaced with ordinary cement (A-10-type or A-20-type), for the purpose of early solidification, solidifies faster than the C-type. Furthermore, with the long-term material age of 28 days or more, the strength is 10 to 11 MN/m^2^ or more in the case of the mixture with a 10% substitution (A-10-type), and approximately 6 to 8 MN/m^2^ in the case of the mixture with a 20% substitution (A-20-type). In both mixtures, a very high strength of 3 to 5 times or more is exhibited as compared with the strength of the C-type.

Comparing the B-type and the C-type shown in Figure 5b, the strength of the B-type is slow to develop, like the A-type, and at the material age of 28 days, the strength is finally equivalent to that of the C-type. After that, the strength development showed a tendency for a rapid increase in strength, which was 10 MN/m^2^ at the material age of 56 days and 13 MN/m^2^ at the material age of 91 days, which is more than 5 to 6 times that of the C-type. In addition, in the case of 10% or 20% replacement with ordinary cement (B-10-type or B-20-type), the strength development is faster than that of the C-type, as with the A-type. Specifically, after the long-term material age of 28 days, the mixture with a 10% substitution (B-10-type) is approximately 7 to 11 MN/m^2^, the mixture with a 20% substitution (B-20-type) is approximately 6 to 8 MN/m^2^, and the higher strength development of 5 times or more than that of the C-type is expected.

In the strength comparison of the A-type and the B-type, shown in Figure 5c, the A-type shows some superiority in terms of early solidification. However, under the same mixing conditions, each improved soil body develops almost the same level of strength after the material age of 56 days. From this, it can be confirmed that the B-type can be applied in the same manner as the A-type based on the fact that there is a slight difference in strength development due to early solidification.

#### 4.3.3. Applicability of Solidifying Material

Through the results of the above-mentioned unconfined compression tests, using sandy soil and cohesive soil as the target soil, it was found that the B-type and the A-type can exhibit higher strength than when using the C-type, especially in cohesive soil. The reason why higher long-term strength develops in cohesive soil is that cohesive soil has a large surface area of soil particles due to its fine particle size and the many contact points between the soil particles, so that the pozzolanic reaction is promoted. Similarly, crystals of calcium silicate hydrate grow fibrously to fill the voids between soil particles and harden [7,8,9,10]. Furthermore, the elution of SiO_2_ and Al_2_O_3_ contained in clay minerals increases the adhesion effect of the silica colloid and increases the strength due to the pozzolanic reaction, resulting in higher strength than in sandy soil [7,8,9,10]. From the characteristics of this solidifying material, which develops higher strength for cohesive soil, it is expected that the design specifications for ground improvement, such as the improved thickness and area, can be made smaller than before, which is a useful point.

In addition, it was found that the B-type showed slower strength development in terms of early solidification than the A-type in both sandy soil and cohesive soil. This is because the reaction ratio differs due to the difference in particle size because the pulverization is not treated in the manufacturing process of the mixing type of ES additive for the purpose of preventing the scattering of the alkaline aid when using the additive. Therefore, if early solidification is required in a field construction, it is desirable to replace the fine powder of blast furnace slag with a small amount of ordinary cement.

Although the usefulness of the solidifying material with the mixing type of ES additive was confirmed in this study, there was concern regarding the elution of harmful substances because the solidifying material with the mixing type of ES additive is mainly composed of inorganic waste and by-products. Therefore, an elution test was conducted according to the “Testing methods for industrial wastewater, JIS K 0102” (JIS 1996) [18]. using a sample of the solidifying material with the mixing type of ES additive coarsely crushed to less than 2 mm. In Table 4, which shows the results of the test, the elution amounts of cadmium, lead, hexavalent chromium, arsenic, total mercury, selenium, fluorine, and boron are seen to be below the environmental standard values, and thus their environmental safety has been confirmed.

## 5. Conclusions

To verify the applicability of the mixture of the mixing type of ES additive and blast furnace slag fine powder as a solidifying material, the performance was compared in some indoor interior mixing tests in this study. From these test results, the following characteristics of the solidifying material using the mixing type of ES additive were clarified.

(1) When mixed with cohesive soil, high fluidity was obtained that could not be obtained with cement-based solidifying materials.
(2) Higher strength can be expected with cohesive soil than with a cement-based solidifying material.
(3) When the solidifying material is used as it is, the strength development is slower than that of the cement-based solidifying material, resulting in a delayed effect. In addition, when trying to attain early solidification, there is a method of partially replacing the fine powder of blast furnace slag with ordinary cement.
(4) Compared with the solidifying material using the heat-treating type of ES additive [7,8,9,10] developed in the past, there is a slight delay in terms of early solidification.

In terms of (1), it is suggested that it is highly applicable to both the mechanical stirring method and the high-pressure injection stirring method. When performing ground-improvement work on cohesive soil, the soil mixed with the solidifying material will have high fluidity. As a result, the mechanical stirring method facilitates stirring, which enables construction with a machine having a small stirring force and improves the stirring efficiency, which leads to an improvement in the quality of the improved soil body. Moreover, with the high-pressure injection stirring method, the quality of the improved soil body is improved by improving the stirring efficiency. In addition, because higher fluidity can be obtained, it is thought that the suppression of blockage due to mud drainage and the influence on nearby structures will be reduced.

In terms of (2), this is a point that it is widely found to be useful in ground improvement. When the solidifying material of this study is compared with the same amount of the conventional cement-based solidifying material, the improved soil body made of cohesive soil has relatively high strength. The amount of solidifying material can be reduced. As a result, it can be used under conditions where the goal is to avoid problems such as construction defects due to a decrease in the fluidity of the target soil brought about by an increase in the amount of solidifying material added. Furthermore, the thickness and width of the portion of the ground that was improved by the design specifications can be reduced in comparison to the conventional construction method.

In terms of (3), it is suggested that the mechanical stirring method is highly applicable to cohesive soil as well as to the above-mentioned (2). Conventionally, in the design specifications whereby the adjacent improved soil columns, improved by the block-type improvement, wall-type improvement or grid-type improvement, overlap and become integrated with each other, if the construction of adjacent improved soil columns is significantly delayed, the columns overlap due to the hardening progress of the preceding improved soil column. In some cases, it is not possible to carry out the construction, but by utilizing the delay effect of this solidifying material, it is possible to handle duplicate construction even with the mechanical stirring method. Another advantage is that it can prevent poor solidification due to the use of retarders. On the other hand, as a method for obtaining early solidification, when a portion of the fine powder of blast furnace slag is replaced with ordinary cement, it is possible for the strength to develop earlier than that of the cement-based solidifying material.

In terms of (4), it is thought that the reaction rate differs depending on the difference in particle size based on the presence or absence of pulverization treatment. However, when focusing on the solidification performance of the additive itself, at present, the authors believe that it can be handled to some extent by partially replacing the above-mentioned method with ordinary cement.

The authors were able to confirm the applicability of the mixing type of ES additive as a solidifying material for ground improvement. In particular, because it has the same characteristics as the heat-treating type of ES additive [7,8,9,10], which can be expected to develop higher strength in cohesive soil, it can be used under conditions that are difficult to handle with cement-based solidifying materials. On the other hand, regarding the delay effect of the solidifying material with the mixing type of ES additive, it is more applicable to the mechanical stirring method than the conventional construction method. However, the high-pressure injection stirring method may have more restrictions. Therefore, it is necessary to consider mixing adjustments assuming early solidification by partial replacement with ordinary cement.

## Figures and Tables

**Table 1:** Conditions of specimen mixing in each interior mixing test.

No.	Solidifying Material							Target Soil
	SL/H = 10 (*)						C-Type	
	A-Type			B-Type				
	A-Type	A-10-Type	A-20-Type	B-Type	B-10-Type	B-20-Type		
1	√							Sandy Soil S/M = 2.0 (*)
2		√						
3			√					
4				√				
5					√			
6						√		
7							√	
8	√							Cohesive soil S/M = 2.0 (*)
9		√						
10			√					
11				√				
12					√			
13						√		
14							√	

(*) SL: fine powder of blast furnace; H: heat-treating type of ES additive or mixing type of ES additive; S: target soil; M: slurry solution.

**Table 2:** Results of fluidity tests using slurry solutions of various solidifying materials.

No.	Solidifying Material							Viscosity by Fluidity Test (cm)	
	SL/H = 10 (*)						C-Type		
	A-Type			B-Type				Immediately After	After 3 h
	A-Type	A-10-Type	A-20-Type	B-Type	B-10-Type	B-20-Type			
1	√							8.7	8.9
2		√						8.9	9.3
3			√					8.9	9.9
4				√				8.5	8.7
5					√			8.8	9.0
6						√		8.8	9.7
7							√	9.0	9.2
8	√							8.7	8.9
9		√						8.9	9.3
10			√					8.9	9.9
11				√				8.5	8.7
12					√			8.8	9.0
13						√		8.8	9.7
14							√	9.0	9.2

(*) SL: fine powder of blast furnace; H: heat-treating type of ES additive or mixing type of ES additive; S: target soil; M: slurry solution.

**Table 3:** Results of the flow tests for the specimen mixed with cohesive soil and the slurry solution of each solidifying material.

No.	Solidifying Material							Flow Value by Flow Test (cm)	Target Soil
	SL/H = 10 (*)						C-Type		
	A-type			B-Type				Immediately After	
	A-Type	A-10-Type	A-20-Type	B-Type	B-10-Type	B-20-Type			
8	√							41.0	Cohesive soil S/M = 2.0 (*)
9		√						27.5	
10			√					20.5	
11				√				43.5	
12					√			34.5	
13						√		26.5	
14							√	11.5	

(*) SL: fine powder of blast furnace; H: heat-treating type of ES additive or mixing type of ES additive; S: target soil; M: slurry solution.

**Table 4:** Results of the elution test for using a sample of the solidifying material with the mixing type of ES additive coarsely crushed to less than 2 mm.

Item	Unit	Test Method	Measured Value
Cadmium	mg/L	JIS K 0102 55.4 (1998)	<0.001
Lead	mg/L	JIS K 0102 54.4 (1998)	0.004
Hexavalent chromium	mg/L	JIS K 0102 65.2.4 (1998)	<0.005
Arsenic	mg/L	JIS K 0102 61.2 (1998)	<0.001
Total mercury	mg/L	Environmental Agency Notification No. 59 (1971)	<0.0005
Selenium	mg/L	JIS K 0102 67.2 (1998)	0.003
Fluorine	mg/L	JIS K 0102 34.1 (1998)	0.3
Boron	mg/L	JIS K 0102 47.3 (1998)	0.17

## Data Availability

Data sharing is applicable to this article.

## References

[1] Mohajerani A., Vajna J., Cheung T.H.H., Kurmus H., Arulrajah A., Horpibulsuk S. (2017). Practical recycling applications of crushed waste glass in construction materials: A review. Constr. Build. Mater..

[2] Khan M.S., Tufail M., Mateeullah M. (2018). Effects of Waste Glass Powder on the Geotechnical Properties of Loose Subsoils. Civ. Eng. J..

[3] Kazmi D., Serati M., Williams D.J., Qasim S., Cheng Y.P. (2020). The potential use of crushed waste glass as a sustainable alternative to natural and manufactured sand in geotechnical applications. J. Clean. Prod..

[4] Ishimura Y., Takeuchi K. (2015). Does conflict matter? Spatial distribution of disposal sites in Japan. Environ. Econ. Policy Stud..

[5] Okubo S., Matsumoto T. (2020). Potential evaluation of transportation efficiency of reverse logistics based on actual data of industrial waste treatment company. J. Hum. Environ. Symbiosis.

[6] Suzuki S., Natori Y., Saito M., Arai K., Takahata O., Akutagawa T., Okada H., Kishimoto A., Fujii K., Takenaka M. (2020). Possible interactions between policy formulations and risk studies. Jpn. J. Risk Anal..

[7] Inazumi S. (2017). Potential for silica-based solidification materials as soil improving agents. Int. J. GEOMATE.

[8] Inazumi S., Intui S., Shinsaka T., Hashimoto R. (2019). Physical Analysis of Solidifying Mechanism for Mixed Solidification Material with Silica Admixture and Blast Furnace Slag. J. Solid Waste Technol. Manag..

[9] Inazumi S., Intui S., Jotisankasa A., Chaiprakaikeow S., Shinsaka T. (2019). Applicability of mixed solidification material based on inorganic waste as soil stabilizer. Case Stud. Constr. Mater..

[10] Inazumi S., Shinsaka T., Hashimoto R., Iwamoto H. (2020). Hardening Mechanism and Time on Silica-Based Solidifying Material as Ground Improvement Material. J. Soc. Mater. Sci. Jpn..

[11] Japan Cement Association (JCA) (2012). Manual on Ground Improvement Using Cement-Based Solidifying Materials.

[12] Terashi M. The State of Practice in Deep Mixing Methods. Proceedings of the Third International Conference on Grouting and Ground Treatment.

[13] Farouk A., Shahien M.M. (2013). Ground improvement using soil–cement columns: Experimental investigation. Alex. Eng. J..

[14] Croce P., Flora A., Modoni G. (2014). Jet Grouting: Technology, Design and Control.

[15] Güllü H. (2015). On the viscous behavior of cement mixtures with clay, sand, lime and bottom ash for jet grouting. Constr. Build. Mater..

[16] Katsumi T., Inazumi S. (2016). Transition of Ground Improvement Technologies in Japan. J. Soc. Mater. Sci. Jpn..

[17] Canakci H., Güllü H., Dwle M.I.K. (2017). Effect of Glass Powder Added Grout for Deep Mixing of Marginal Sand with Clay. Arab. J. Sci. Eng..

[18] JIS (1996). JIS K 0102 1996. Testing Methods for Industrial Wastewater Standard by JIS/Japanese Standard Association.

